# Parameter and State Estimator for State Space Models

**DOI:** 10.1155/2014/106505

**Published:** 2014-03-02

**Authors:** Ruifeng Ding, Linfan Zhuang

**Affiliations:** ^1^Key Laboratory of Advanced Process Control for Light Industry (Ministry of Education), Jiangnan University, Wuxi 214122, China; ^2^School of Internet of Things Engineering, Jiangnan University, Wuxi 214122, China

## Abstract

This paper proposes a parameter and state estimator for canonical state space systems from measured input-output data. The key is to solve the system state from the state equation and to substitute it into the output equation, eliminating the state variables, and the resulting equation contains only the system inputs and outputs, and to derive a least squares parameter identification algorithm. Furthermore, the system states are computed from the estimated parameters and the input-output data. Convergence analysis using the martingale convergence theorem indicates that the parameter estimates converge to their true values. Finally, an illustrative example is provided to show that the proposed algorithm is effective.

## 1. Introduction

Parameter estimation and identification have had important applications in system modelling, system control, and system analysis [1–5] and thus have received much research attention in recent decades [6–11]. Several identification methods have been developed for state space models, for example, the subspace identification methods [12]. Gibson and Ninness presented a robust maximum-likelihood estimation for fully parameterized linear time-invariant (LTI) state space models; the idea is to use the expectation maximization (EM) algorithm to estimate maximum-likelihood degrees [13]. Raghavan et al. studied the EM-based state space model identification problems with irregular output sampling [14].

The state space model includes not only the unknown parameter matrices/vectors, but also the unknown noise terms in the formation vector and unmeasurable state vector. Many algorithms can estimate the system states assuming that the system parameter matrices/vectors are available but such state estimation algorithm cannot work if the system parameters are unknown [15]. Recently, Ding presented a combined state and least squares parameter estimation algorithm for dynamic systems [16].

In the area of state space model identification, Ding and Chen proposed a hierarchical identification estimation algorithm for estimating the system parameters and states [17]. Li et al. assumed that the system states were available and used the measurable states and input-output data to estimate the parameters of lifted state space models for general dual-rate systems [18]. Recently, some identification methods have been developed, for example, the least squares methods [19, 20], the gradient-based methods [21, 22], the bias compensation methods [23, 24], and the maximum likelihood methods [25–30]. The objective of this paper is to present a new parameter and state estimation-based residual algorithm from the given input-output data and further to analyze the convergence of the proposed algorithm.

The convergence analysis of identification algorithms has always been one of the important projects in the field of control. By using the stochastic martingale theory, Ding et al. studied the properties of stochastic gradient identification algorithms under weak conditions [31]. Ding and Liu discussed the gradient-based identification approach and convergence for multivariable systems with output measurement noise [32]. Other identification methods for linear or nonlinear systems [33–42] include the auxiliary model identification methods [43–57], the hierarchical identification methods [58–73], and the two-stage or multistage identification methods [74–78].

This paper is organized as follows.  Section 2 introduces the system description and its identification model paper.  Section 3 derives a basic parameter identification algorithm for canonical state space systems and analyzes the performance of the proposed algorithm.  Section 4 gives a state estimation algorithm.  Section 5 provides an example for the proposed algorithm. Finally, concluding remarks are given in Section 6.

## 2. System Description and Identification Model

Let us introduce some notation [15]. “*A* = :*X*” or “*X* : = *A*” stands for “*A* is defined as *X*”; the symbol **I**(**I**_*n*_) stands for an identity matrix of appropriate size (*n* × *n*); the superscript T denotes the matrix transpose; |**X**| = det⁡[**X**] represents the determinant of a square matrix **X**; the norm of a matrix **X** is defined by ||**X**||^2^ = tr⁡[**X** **X** ^T^]; 1_*n*_ : = 1_*n*×1_ represents an *n* × 1 vector whose elements are all 1; *λ*_min⁡_[**X**] represents the minimum eigenvalues of **X**; for *g*(*t*)⩾0, we write *f*(*t*) = *O*(*g*(*t*)) if there exists a positive constant *δ*_1_ such that |*f*(*t*)| ⩽ *δ*_1_ *g*(*t*).

In order to study the convergence of the algorithm proposed in [15], here we simply give that algorithm in [15]. Consider a linear system described by the following observability canonical state space model [15]:

(1) x(t+1)=Ax(t)+bu(t),y(t)=cx(t)+v(t),

(2) A:=[010⋯0001⋱⋮⋮⋮⋱000⋯01−an−an−1−an−2⋯−a1]∈ℝn×n,b:=[b1b2⋮bn]∈ℝn,c:=[1,0,0,…,0]∈ℝ1×n,

where **x**(*t*) ∈ ℝ^*n*^ is the state vector, *u*(*t*) ∈ ℝ is the system input, *y*(*t*) ∈ ℝ is the system output, and *v*(*t*) ∈ ℝ is a random noise with zero mean. Assume that the order *n* is known, and *u*(*t*) = 0, *y*(*t*) = 0 and *v*(*t*) = 0 for *t* ⩽ 0.

The system in (1) is an observability canonical form, and its observability matrix **Q**_*o*_ is an identity matrix; that is,

(3) Qo:=[ccA⋮cAn−1]=In.

For the system in (1), the objective of this paper is to develop a new algorithm to estimate the parameter matrix/vector **A** and **b** (i.e., the parameters *a*_*i*_ and *b*_*i*_) and the system state vector **x**(*t*) from the available measurement input-output data {*u*(*t*), *y*(*t*)}.

Since the available measurement input-output data {*u*(*t*), *y*(*t*)} are known but the state vector **x**(*t*) is unknown, it is required to eliminate the state vector from (1) and obtain a new expression which only involves the input and output, in order to obtain the estimates of the parameters in (1). The following derives the identification model based on the method in [15].

Define some vectors/matrix,

(4) φy(t):=[y(t−n),y(t−n+1),…,y(t−1)]T∈ℝn,φu(t):=[u(t−n),u(t−n+1),…,u(t−1)]T∈ℝn,φv(t):=[v(t−n),v(t−n+1),…,v(t−1)]T∈ℝn,M:=[00⋯00cb0⋯00cAbcb⋱⋮⋮⋮⋮⋱00cAn−2bcAn−3b⋯cb0]∈ℝn×n.

From (1), we have

(5) y(t)=cx(t)+v(t),

(6) y(t+1)=cx(t+1)+v(t+1)=c[Ax(t)+bu(t)]+v(t+1)=cAx(t)+cbu(t)+v(t+1),

(7) y(t+2)=cAx(t+1)+cbu(t+1)+v(t+2)=cA[Ax(t)+bu(t)]+cbu(t+1)+v(t+2)=cA2x(t)+cAbu(t)+cbu(t+1)+v(t+2), ⋮

(8) y(t+n−1)=cAn−1x(t)+cAn−2bu(t)+cAn−3bu(t−1) +⋯+cbu(t+n−2)+v(t+n−1),

(9) y(t+n)=cAnx(t)+cAn−1bu(t)+cAn−2bu(t−1) +⋯+cbu(t+n−1)+v(t+n).

Combining (5) with (8) gives

(10) φy(t+n)=Qox(t)+Mφu(t+n)+φv(t+n)=x(t)+Mφu(t+n)+φv(t+n),

or

(11) x(t)=φy(t+n)−Mφu(t+n)−φv(t+n).

Define the parameter vector ***θ*** and the information vector **φ**(*t*) as

(12) θ:=[θaθb]∈ℝ2n,θa:=[cAn]T∈ℝn,θb:=[−cAnM+[cAn−1b,cAn−1b,…,cb]]T∈ℝn,φ(t+n):=[φyT(t+n)−φvT(t+n),φuT(t+n)]T∈ℝ2n.

Substituting (11) into (9) gives

(13) y(t+n) =cAn[φy(t+n)−Mφu(t+n)−φv(t+n)]  +cAn−1bu(t)+cAn−2bu(t−1)  +⋯+cbu(t+n−1)+v(t+n) =cAn[φy(t+n)−Mφu(t+n)−φv(t+n)]  +[cAn−1b,cAn−2b,…,cb][u(t)u(t−1)⋮u(t+n−1)]+v(t+n) =cAn[φy(t+n)−φv(t+n)]−cAnMφu(t+n)  +[cAn−1b,cAn−2b,…,cb]φu(t+n)+v(t+n) =[φyT(t+n)−φvT(t+n),φuT(t+n)][θaθb]+v(t+n) =φT(t+n)θ+v(t+n).

Replacing *t* in (13) with *t* − *n* yields

(14) y(t)=φT(t)θ+v(t),

which is called the identification model or identification expression of the state-space model.

## 3. The Parameter Estimation Algorithm and Its Convergence

The recursive least squares algorithm for estimating ***θ*** is expressed as

(15) θ^(t)=θ^(t−1)+P(t)φ^(t)[y(t)−φ^T(t)θ^(t−1)],

(16) P−1(t)=P−1(t−1)+φ^(t)φ^T(t),  P(0)=p0I,

(17) v^(t)=y(t)−φ^T(t)θ^(t),

(18) φ^(t)=[y(t−n)−v^(t−n),  y(t−n+1)−v^(t−n+1),…,  y(t−1)−v^(t−1),u(t−n),  u(t−n+1),…,u(t−1)]T.

This algorithm is commonly used for convergence analysis. To avoid computing the matrix inversion, this algorithm is equivalently expressed as

(19) θ^(t)=θ^(t−1)+L(t)[y(t)−φ^T(t)θ^(t−1)],

(20) L(t)=P(t)φ^(t)=P(t−1)φ^(t)1+φ^T(t)P(t−1)φ^(t),

(21) P(t)=[I−L(t)φ^T(t)]P(t−1),  P(0)=p0I,

(22) v^(t)=y(t)−φ^T(t)θ^(t),

(23) φ^(t)=[φ^T(t−n)θ^(t−n),  φ^T(t−n+1)θ^(t−n+1),…,φ^T(t−1)θ^(t−1),  u(t−n),u(t−n+1),…,u(t−1)]T,

where **L**(*t*) ∈ ℝ^2*n*^ is the gain vector.

Define the parameter estimation error vector $\tilde{\theta }(t):=\;\hat{\theta }(t)-\theta$ and the nonnegative function $T(t):={\tilde{\theta }}^{\text{T}}\left(t\right)P^{-1}\left(t\right)\tilde{\theta }\left(t\right)$ .

**Theorem 1**

For the system in (1) and algorithm in (15)–(18), assume that {*v*(*t*), *ℱ*_*t*_} is a martingale difference sequence defined on a probability space {*Ω*, *ℱ*, *P*}, where {*ℱ*_*t*_} is the *σ* algebra sequence generated by the observations up to and including time *t*. The noise sequence {*v*(*t*)} satisfies the following assumptions:

(A1) E [*v*(*t*) | *ℱ*_*t*−1_] = 0, a.s.,
(A2) E [*v* ^2^(*t*) | *ℱ*_*t*−1_] ⩽ *σ* ^2^ < *∞*, a.s.,
(A3) *A*′(*z*): = *A* ^−1^(*z*) − 1/2 is strictly positive real.

Then the following inequality holds:

(24)
E
[T(t)+S(t) ∣ ℱt−1]  ⩽T(t−1)+S(t−1)+2φ^
T
(t)P(t)φ^(t)σ2,

where

(25) S(t):=2∑i=1tu~(i)y~(i)⩾0,

(26) u~(t):=−φ^
T
(t)θ~(t),

(27) y~(t):=12φ^
T
(t)θ~(t)+[y(t)−φ^
T
(t)θ^(t)−v(t)].

**Proof**

Define the innovation vector $e(t):=y(t)-\;{\hat{\varphi }}^{\text{T}}\left(t\right)\hat{\theta }(t-1)$ . Using (17), it follows that

(28) v^(t)=[1−φ^T(t)P(t)φ^(t)]e(t)=e(t)1+φ^T(t)P(t−1)φ^(t).

Subtracting ***θ*** from both sides of (15) and using (14), we have

(29) θ~(t)=θ^(t)−θ=θ~(t−1)+P(t)φ^(t)e(t)=θ~(t−1)+P(t−1)φ^(t)v^(t).

According to the definition of *T*(*t*) and using (16) and (29), we have

(30) T(t)=T(t−1)+θ~T(t)φ^(t)φ^T(t)θ~(t) +2φ^T(t)θ~(t)v^(t)−φ^T(t)P(t)φ^(t) ×[1−φ^T(t)P(t)φ^(t)]e2(t)⩽T(t−1)+θ~T(t)φ^(t)φ^T(t)θ~(t) +2φ^T(t)θ~(t)v^(t)=T(t−1)+2φ^T(t)θ~(t) ×[12θ~T(t)φ^(t)+(v^(t)−v(t))]+2φ^T(t)θ~(t)v^(t).

Using (26), (27), and (29), and $0⩽{\hat{\varphi }}^{\text{T}}\left(t\right)P\left(t\right)\hat{\varphi }(t)⩽1$ , we have

(31) T(t)⩽T(t−1)−2u~(t)y~(t)+2φ^T(t) ×[θ~(t−1)+P(t)φ^(t)e(t)]v(t)=T(t−1)−2u~(t)y~(t)+2φ^T(t)θ~(t−1)v(t) +2φ^T(t)P(t)φ^(t)[e(t)−v(t)]v(t)+v2(t).

Since ${\hat{\varphi }}^{\text{T}}\left(t\right)\tilde{\theta }\left(t-1\right)$ , *e*(*t*) − *v*(*t*), ${\hat{\varphi }}^{\text{T}}\left(t\right)P\left(t\right)\hat{\varphi }(t)$ are uncorrelated with *v*(*t*) and are *ℱ*_*t*−1_-measurable, taking the conditional expectation with respect to *ℱ*_*t*−1_ and using (A1)-(A2) give

(32) E[T(t) ∣ ℱt−1]⩽T(t−1)−2E[u~(t)y~(t) ∣ ℱt−1] +2φ^T(t)P(t)φ^(t)σ2, a.s.

The state space model in (1) can be transformed into an input-output representation,

(33) y(t)=c(zI  −A)−1bu(t)+v(t)=c adj[zI−A]bdet⁡⁡[zI−A]u(t)+v(t)=:B(z)A(z)u(t)+v(t),

where adj[*z* **I** − **A**] is the adjoint matrix of [*z* **I** − **A**], *A*(*z*) and *B*(*z*) are polynomials in a unit backward shift operator *z* ^−1^[*z* ^−1^ *y*(*t*) = *y*(*t* − 1)], and

(34) A(z)≔z−ndet⁡[zI−A],B(z):=z−nc adj[zI−A]b.

Referring to the proof of Lemma 3 in [43], using (33), we have

(35) A(z)[v^(t)−v(t)]=A(z)v^(t)−A(z)y(t)+B(z)u(t)=−A(z)φ^T(t)θ^(t)+B(z)u(t)=−φ^T(t)θ~(t)=u~(t).

Using (17), (26), and (35), from (27), we get

(36) y~(t)=12φ^T(t)θ~(t)+[v^(t)−v(t)]=[A−1(z)−12]u~(t).

Since *A*′(*z*) is a strictly positive real function, referring to Appendix C in [79], we can obtain the conclusion *S*(*t*)⩾0. Adding both sides of (32) by *S*(*t*) gives the conclusion of Theorem 1.

**Theorem 2**

For the system in (1) and the algorithm in (15)–(18), assume that (A1)–(A3) hold and that *A*(*z*) is stable; that is, all zeros of *A*(*z*) are inside the unit circle; then the parameter estimation error satisfies

(37)   ||θ^(t)−  θ||2=O([ln⁡r(t)]cλmin⁡[P−1(t)]), a.s.,  for  any  c>1.

**Proof**

Using the formula *λ*_min⁡_[**Q**]||**x**||^2^ ⩽ **x** ^T^ **Q** **x** ⩽ *λ*_max⁡_[**Q**]||**x**||^2^, and from the definition of *T*(*t*), we have

(38) ||θ~(t)||2⩽θ~T(t)P−1(t)θ~(t)λmin⁡[P−1(t)]=T(t)λmin⁡[P−1(t)].

Let

(39) W(t):=T(t)+S(t)[ln⁡|P−1(t)|]c, c>1.

Since ln⁡|**P** ^−1^(*t*)| is nondecreasing, using Theorem 1 yields

(40) E[W(t) ∣ ℱt−1]⩽T(t−1)+S(t−1)[ln⁡|P−1(t)|]c+2φ^T(t)P(t)φ^(t)[ln⁡|P−1(t)|]cσ2⩽V(t−1)+2φ^T(t)P(t)φ^(t)[ln⁡|P−1(t)|]cσ2, a.s.

Referring to the proof of Theorem 2 in [43], we have

(41) ||θ~(t)−θ||2=O([ln⁡|P−1(t)|]cλmin⁡[P−1(t)])=O([ln⁡r(t)]cλmin⁡[P−1(t)]), a.s.  for  any  c>1.

Assume that there exist positive constants *γ*, *c*_1_, *c*_2_, and *t*_0_ such that the following generalized persistent excitation condition (unbounded condition number) holds:

(42) c1I⩽1t∑j=1tφ(j)φT(j)⩽c2tγI, a.s.,  for  t⩾t0.

Then for any *c* > 1, we have

(43) ||θ^(t)−θ||2=O([ln⁡t]ct)⟶0, a.s.  for  any  c>1.

## 4. The State Estimation Algorithm

Referring to the method in [15], the state estimate $\hat{x}(t)$ of the state vector **x**(*t*) can be expressed as

(44) x^(t−n)=φy(t)−M^(t)φu(t)−φ^v(t),

(45) φy(t)=[y(t−n),y(t−n+1),…,y(t−1)]T,

(46) φu(t)=[u(t−n),u(t−n+1),…,u(t−1)]T,

(47) φ^v(t)=[v^(t−n),v^(t−n+1),…,v^(t−1)]T,

(48) M^(t)=[00⋯00b^1(t)0⋯00b^2(t)b^1(t)⋱⋮⋮⋮⋮⋱00b^n−1(t)b^n−2(t)⋯b^1(t)0],

(49) [b^1(t)b^2(t)⋮b^n−1(t)b^n(t)]=[a^n−1(t)a^n−2(t)⋯a^1(t)1a^n−2(t)a^n−3(t)⋯10⋮⋮⋮⋮a^1(t)1⋯0010⋯00]−1θ^b(t),

(50) θ^(t)=[θ^a(t)θ^b(t)],

(51) θ^a(t)=[−a^n(t),−a^n−1(t),…,−a^1(t)]T.

To summarize, we list the steps involved in the algorithm in (19)–(23) and (44)–(51) to compute the parameter estimate $\hat{\theta }(t)$ and the state estimate $\hat{x}(t-n)$ .

1. Let *t* = 1; set the initial values $\hat{\theta }(i)=1_{n}/p_{0}$ , **P**(0) = *p*_0_ **I**, *u*(*i*) = 0, *y*(*i*) = 0, $\hat{v}(i)=0$ , or $\hat{v}(i)=1/p_{0}$ for *i* ⩽ 0, *p*_0_ = 10^6^. Give a small positive number *ε*.
2. Collect the input-output data *u*(*t*) and *y*(*t*); form $\hat{\varphi }(t)$ using (23), **φ**_*y*_(*t*) using (45), and **φ**_*u*_(*t*) using (46).
3. Compute the gain vector **L**(*t*) using (20) and the covariance matrix **P**(*t*) using (21).
4. Update the parameter estimation vector $\hat{\theta }(t)$ using (19).
5. Compute $\hat{v}(t)$ using (22), and form ${\hat{\varphi }}_{v}(t)$ using (47).
6. Determine ${\hat{a}}_{i}(t)$ using (51) and compute ${\hat{b}}_{i}(t)$ using (49); then form $\hat{M}(t)$ using (48).
7. Compute the state estimate $\hat{x}(t-n)$ using (44).
8. If they are sufficiently close, if $||\hat{\theta }(t)-\hat{\theta }\left(t-1\right)||⩽\varepsilon$ , then terminate the procedure and obtain the estimate $\hat{\theta }(t)$ ; otherwise, increase *t* by 1 and go to step 2.

## 5. Example

Consider the following single-input single-output second-order system in canonical form:

(52) x(t+1)=[01−0.701.35]x(t)+[11]u(t),y(t)=[1, 0]x(t)+v(t).

**Figure 1:**
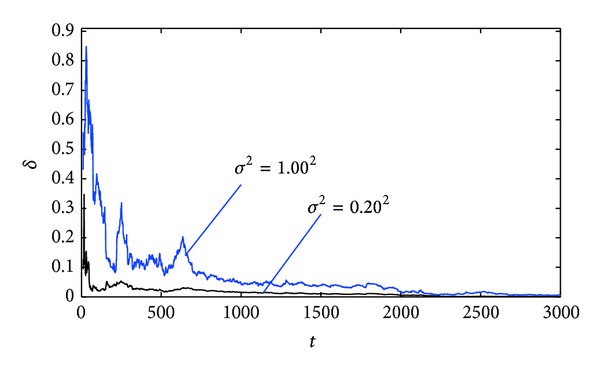
The parameter estimation errors *δ* versus *t* (*σ* ^2^ = 0.20^2^ and *σ* ^2^ = 1.00^2^).

**Figure 2:**
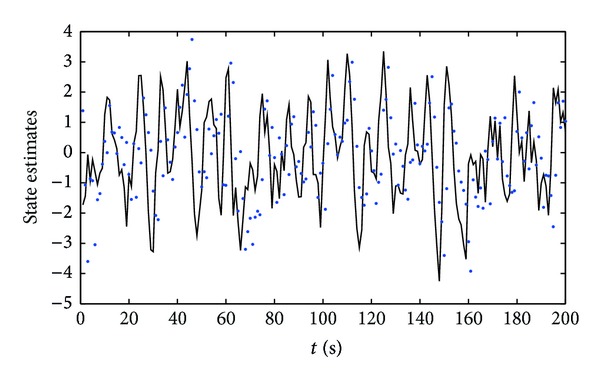
The state estimation errors *δ* versus *t* (*σ* ^2^ = 0.20^2^). Solid line: the true *x*_1_(*t*); dots: the estimated ${\hat{x}}_{1}(t)$ .

**Figure 3:**
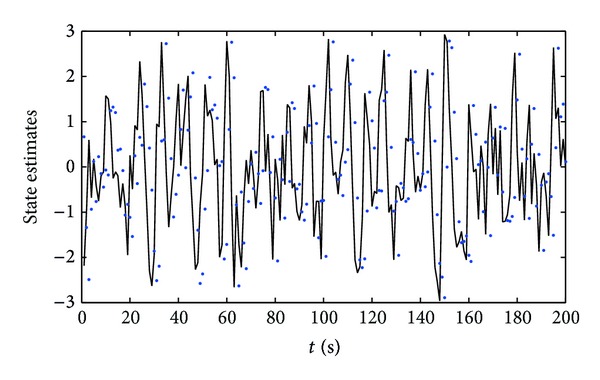
The state estimation errors *δ* versus *t* (*σ* ^2^ = 0.20^2^). Solid line: the true *x*_2_(*t*); dots: the estimated ${\hat{x}}_{2}(t)$ .

The simulation conditions are the same as in [15]. That is, the input {*u*(*t*)} is taken as an independent persistent excitation signal sequence with zero mean and unit variances and {*v*(*t*)} as a white noise sequence with zero mean and variances *σ* ^2^ = 0.20^2^ and *σ* ^2^ = 1.00^2^, respectively. Apply the proposed parameter and state estimation algorithm in (19)–(23) and (44)–(51) to estimate the parameters and states of this example system; the parameter estimates and their estimation errors are shown in Tables 1 and 2; the parameter estimation errors *δ* versus *t* are shown in Figure 1; the states *x*_*i*_(*t*) and their estimates ${\hat{x}}_{i}(t)$ versus *t* are shown in Figures 2 and 3, where $\delta :=||\hat{\theta }(t)-\theta ||/||\theta ||$ (||**x**||^2^ = **x** ^T^ **x**) is the parameter estimation error.

From the simulation results of Tables 1 and 2 and Figures 1–3, we can draw the following conclusions.

1. A lower noise level leads to a faster rate of convergence of the parameter estimates to the true parameters.
2. The parameter estimation errors *δ* become smaller (in general) as the data length *t* increases; see Tables 1 and 2 and Figure 1. In other words, increasing data length generally results in smaller parameter estimation errors.
3. The state estimates are close to their true values with *t* increasing; see Figures 2 and 3. These indicate that the proposed parameter and state estimation algorithm are effective.

## 6. Conclusions

In this paper, the identification problems for linear systems based on the canonical state space models with unknown parameters and states are studied. A new parameter and state estimation algorithm has been presented directly from input-output data. The analysis using the martingale convergence theorem indicates that the proposed algorithms can give consistent parameter estimation. The simulation results show that the proposed algorithms are effective. The method in this paper can combine the multiinnovation identification methods [80–92], the iterative identification methods [93–100], and other identification methods [101–111] to present new identification algorithms or to study adaptive control problems for linear or nonlinear, single-rate or dual-rate, scalar or multivariable systems [112–117].

## Figures and Tables

**Table 1:** The parameter estimates and errors (σ^2^ = 0.20^2^).

*t*	θ_1_	θ_2_	θ_3_	θ_4_	δ (%)
100	−0.70568	1.35339	−0.39169	1.01645	2.44445
200	−0.71515	1.38650	−0.41330	1.00994	4.06207
500	−0.70678	1.36377	−0.38556	0.99926	2.08997
1000	−0.70624	1.36483	−0.37257	1.00253	1.50180
2000	−0.70481	1.35720	−0.35450	0.99836	0.53395
3000	−0.70098	1.35107	−0.34969	0.99994	0.08005

True values	−0.70000	1.35000	−0.35000	1.00000	

**Table 2:** The parameter estimates and errors (σ^2^ = 1.00^2^).

*t*	θ_1_	θ_2_	θ_3_	θ_4_	δ (%)
100	−0.32642	0.84623	−0.03136	1.05653	38.07875
200	−0.60245	1.38498	−0.53220	1.01317	11.33189
500	−0.72060	1.42162	−0.52967	0.98306	10.53486
1000	−0.70654	1.38791	−0.42136	1.00912	4.40155
2000	−0.71358	1.37357	−0.35605	0.98820	1.63287
3000	−0.70120	1.35114	−0.34033	0.99742	0.54719

True values	−0.70000	1.35000	−0.35000	1.00000	

## References

[1] Ding F (2013). *System Identification—New Theory and Methods*.

[2] Ding F (2014). *System Identification—Performances Analysis for Identification Methods*.

[3] Hu YB (2013). Iterative and recursive least squares estimation algorithms for moving average systems. *Simulation Modelling Practice and Theory*.

[4] Wang ZY, Shen YX, Ji ZC, Ding F (2013). Filtering based recursive least squares algorithm for Hammerstein FIR-MA systems. *Nonlinear Dynamics*.

[5] Singh V (2011). Stability of discrete-time systems joined with a saturation operator on the statespace: generalized form of Liu-Michel’s criterion. *Automatica*.

[6] Xiong XL, Fan W, Ding R (2012). Least-squares parameter estimation algorithm for a class of input nonlinear systems. *Journal of Applied Mathematics*.

[7] Shi Y, Fang H (2010). Kalman filter-based identification for systems with randomly missing measurements in a network environment. *International Journal of Control*.

[8] Shi Y, Yu B (2009). Output feedback stabilization of networked control systems with random delays modeled by Markov chains. *IEEE Transactions on Automatic Control*.

[9] Shi Y, Yu B (2011). Robust mixed *H*
_2_/*H*
_
*∞*
_ control of networked control systems with random time delays in both forward and backward communication links. *Automatica*.

[10] Hu PP, Ding F (2013). Multistage least squares based iterative estimation for feedback nonlinear systems with moving average noises using the hierarchical identification principle. *Nonlinear Dynamics*.

[11] Hu PP, Ding F, Sheng J (2013). Auxiliary model based least squares parameter estimation algorithm for feedback nonlinear systems using the hierarchical identification principle. *Journal of the Franklin Institute—Engineering and Applied Mathematics*.

[12] Viberg M (1995). Subspace-based methods for the identification of linear time-invariant systems. *Automatica*.

[13] Gibson S, Ninness B (2005). Robust maximum-likelihood estimation of multivariable dynamic systems. *Automatica*.

[14] Raghavan H, Tangirala AK, Gopaluni RB, Shah SL (2006). Identification of chemical processes with irregular output sampling. *Control Engineering Practice*.

[15] Zhuang L, Pan F, Ding F (2012). Parameter and state estimation algorithm for single-input single-output linear systems using the canonical state space models. *Applied Mathematical Modelling*.

[16] Ding F (2014). Combined state and least squares parameter estimation algorithms for dynamic systems. *Applied Mathematical Modelling*.

[17] Ding F, Chen T (2005). Hierarchical identification of lifted state-space models for general dual-rate systems. *IEEE Transactions on Circuits and Systems I: Regular Papers*.

[18] Li D, Shah SL, Chen T (2001). Identification of fast-rate models from multirate data. *International Journal of Control*.

[19] Ljung L (1999). *System Identification: Theory for the User*.

[20] Xiao Y, Ding F, Zhou Y, Li M, Dai J (2008). On consistency of recursive least squares identification algorithms for controlled auto-regression models. *Applied Mathematical Modelling*.

[21] Ding F, Liu XM, Chen HB, Yao GY (2014). Hierarchical gradient based and hierarchical least squares based iterative parameter identification for CARARMA systems. *Signal Processing*.

[22] Ding F, Chen T (2005). Hierarchical gradient-based identification of multivariable discrete-time systems. *Automatica*.

[23] Zhang Y (2011). Unbiased identification of a class of multi-input single-output systems with correlated disturbances using bias compensation methods. *Mathematical and Computer Modelling*.

[24] Zhang Y, Cui G (2011). Bias compensation methods for stochastic systems with colored noise. *Applied Mathematical Modelling*.

[25] Wang W, Li J, Ding RF (2011). Maximum likelihood parameter estimation algorithm for controlled autoregressive autoregressive models. *International Journal of Computer Mathematics*.

[26] Wang W, Ding F, Dai J (2012). Maximum likelihood least squares identification for systems with autoregressive moving average noise. *Applied Mathematical Modelling*.

[27] Li J, Ding F (2011). Maximum likelihood stochastic gradient estimation for Hammerstein systems with colored noise based on the key term separation technique. *Computers and Mathematics with Applications*.

[28] Li J, Ding F, Yang GW (2012). Maximum likelihood least squares identification method for input nonlinear finite impulse response moving average systems. *Mathematical and Computer Modelling*.

[29] Li JH (2013). Parameter estimation for Hammerstein CARARMA systems based on the Newton iteration. *Applied Mathematics Letters*.

[30] Li JH, Ding F, Hua L (2014). Maximum likelihood Newton recursive and the Newton iterative estimation algorithms for Hammerstein CARAR systems. *Nonlinear Dynamics*.

[31] Ding F, Yang H, Liu F (2008). Performance analysis of stochastic gradient algorithms under weak conditions. *Science in China F*.

[32] Ding F, Liu X-P (2010). Auxiliary model-based stochastic gradient algorithm for multivariable output error systems. *Acta Automatica Sinica*.

[33] Wang DQ, Ding F (2011). Least squares based and gradient based iterative identification for Wiener nonlinear systems. *Signal Processing*.

[34] Wang DQ, Ding F, Chu YY (2013). Data filtering based recursive least squares algorithm for Hammerstein systems using the key-term separation principle. *Information Sciences*.

[35] Wang DQ, Ding F (2012). Hierarchical least squares estimation algorithm for Hammerstein-Wiener systems. *IEEE Signal Processing Letters*.

[36] Ding F, Chen T (2005). Identification of Hammerstein nonlinear ARMAX systems. *Automatica*.

[37] Ding F, Shi Y, Chen T (2006). Gradient-based identification methods for hammerstein nonlinear ARMAX models. *Nonlinear Dynamics*.

[38] Wang DQ, Ding F (2008). Extended stochastic gradient identification algorithms for Hammerstein-Wiener ARMAX systems. *Computers and Mathematics with Applications*.

[39] Wang DQ, Ding F, Liu XM (2014). Least squares algorithm for an input nonlinear system with a dynamic subspace state space model. *Nonlinear Dynamics*.

[40] Wang DQ, Shan T, Ding R (2013). Data filtering based stochastic gradient algorithms for multivariable CARAR-like systems. *Mathematical Modelling and Analysis*.

[41] Wang DQ, Ding F, Zhu DQ (2013). Data filtering based least squares algorithms for multivariable CARAR-like systems. *International Journal of Control, Automation, and Systems*.

[42] Ding F, Liu XP, Liu G (2011). Identification methods for Hammerstein nonlinear systems. *Digital Signal Processing*.

[43] Ding F, Chen T (2004). Combined parameter and output estimation of dual-rate systems using an auxiliary model. *Automatica*.

[44] Ding F, Chen T (2005). Parameter estimation of dual-rate stochastic systems by using an output error method. *IEEE Transactions on Automatic Control*.

[45] Ding F, Shi Y, Chen T (2007). Auxiliary model-based least-squares identification methods for Hammerstein output-error systems. *Systems and Control Letters*.

[46] Ding F, Ding J (2010). Least-squares parameter estimation for systems with irregularly missing data. *International Journal of Adaptive Control and Signal Processing*.

[47] Ding F, Chen T (2004). Identification of dual-rate systems based on finite impulse response models. *International Journal of Adaptive Control and Signal Processing*.

[48] Ding F, Gu Y (2012). Performance analysis of the auxiliary model based least squares identification algorithm for one-step state delay systems. *International Journal of Computer Mathematics*.

[49] Ding F, Gu Y (2013). Performance analysis of the auxiliary model-based stochastic gradient parameter estimation algorithm for state space systems with one-step state delay. *Circuits, Systems and Signal Processing*.

[50] Wang DQ, Chu Y, Yang GW, Ding F (2010). Auxiliary model based recursive generalized least squares parameter estimation for Hammerstein OEAR systems. *Mathematical and Computer Modelling*.

[51] Wang DQ, Chu Y, Ding F (2010). Auxiliary model-based RELS and MI-ELS algorithm for Hammerstein OEMA systems. *Computers and Mathematics with Applications*.

[52] Han LL, Sheng J, Ding F, Shi Y (2009). Auxiliary model identification method for multirate multi-input systems based on least squares. *Mathematical and Computer Modelling*.

[53] Han LL, Wu F, Sheng J, Ding F (2012). Two recursive least squares parameter estimation algorithms for multirate multiple-input systems by using the auxiliary model. *Mathematics and Computers in Simulation*.

[54] Gu Y, Ding F (2012). Auxiliary model based least squares identification method for a state space model with a unit time-delay. *Applied Mathematical Modelling*.

[55] Chen J, Ding F (2012). Least squares and stochastic gradient parameter estimation for multivariable nonlinear Box-Jenkins models based on the auxiliary model and the multi-innovation identification theory. *Engineering Computations*.

[56] Chen J, Zhang Y, Ding RF (2013). Gradient-based parameter estimation for input nonlinear systems with ARMA noises based on the auxiliary model. *Nonlinear Dynamics*.

[57] Chen J, Zhang Y, Ding RF (2010). Auxiliary model based multi-innovation algorithms for multivariable nonlinear systems. *Mathematical and Computer Modelling*.

[58] Ding F, Chen T (2005). Hierarchical least squares identification methods for multivariable systems. *IEEE Transactions on Automatic Control*.

[59] Ding F, Qiu L, Chen T (2009). Reconstruction of continuous-time systems from their non-uniformly sampled discrete-time systems. *Automatica*.

[60] Ding J, Ding F, Liu XP, Liu G (2011). Hierarchical least squares identification for linear SISO systems with dual-rate sampled-data. *IEEE Transactions on Automatic Control*.

[61] Wang L, Ding F, Liu PX (2007). Convergence of HLS estimation algorithms for multivariable ARX-like systems. *Applied Mathematics and Computation*.

[62] Han H, Xie L, Ding F, Liu X (2010). Hierarchical least-squares based iterative identification for multivariable systems with moving average noises. *Mathematical and Computer Modelling*.

[63] Liu YJ, Ding F, Shi Y (2012). Least squares estimation for a class of non-uniformly sampled systems based on the hierarchical identification principle. *Circuits, Systems and Signal Processing*.

[64] Zhang Z, Ding F, Liu X (2011). Hierarchical gradient based iterative parameter estimation algorithm for multivariable output error moving average systems. *Computers and Mathematics with Applications*.

[65] Wang DQ, Ding R, Dong XZ (2012). Iterative parameter estimation for a class of multivariable systems based on the hierarchical identification principle and the gradient search. *Circuits, Systems and Signal Processing*.

[66] Ding F, Chen T (2006). On iterative solutions of general coupled matrix equations. *SIAM Journal on Control and Optimization*.

[67] Ding F, Liu PX, Ding J (2008). Iterative solutions of the generalized Sylvester matrix equations by using the hierarchical identification principle. *Applied Mathematics and Computation*.

[68] Ding F, Chen T (2005). Gradient based iterative algorithms for solving a class of matrix equations. *IEEE Transactions on Automatic Control*.

[69] Ding F, Chen T (2005). Iterative least-squares solutions of coupled Sylvester matrix equations. *Systems and Control Letters*.

[70] Ding F (2010). Transformations between some special matrices. *Computers and Mathematics with Applications*.

[71] Xie L, Ding J, Ding F (2009). Gradient based iterative solutions for general linear matrix equations. *Computers and Mathematics with Applications*.

[72] Ding J, Liu YJ, Ding F (2010). Iterative solutions to matrix equations of the form *A*
_
*i*
_
*XB*
_
*i*
_ = *F*
_
*i*
_. *Computers and Mathematics with Applications*.

[73] Xie L, Liu YJ, Yang H (2010). Gradient based and least squares based iterative algorithms for matrix equations *A*
*XB* + *CX*
^
*T*
^
*D* = *F*. *Applied Mathematics and Computation*.

[74] Ding F (2013). Two-stage least squares based iterative estimation algorithm for CARARMA system modeling. *Applied Mathematical Modelling*.

[75] Ding F, Duan HH (2013). Two-stage parameter estimation algorithms for Box-Jenkins systems. *IET Signal Processing*.

[76] Duan H, Jia J, Ding RF (2012). Two-stage recursive least squares parameter estimation algorithm for output error models. *Mathematical and Computer Modelling*.

[77] Yao G, Ding RF (2012). Two-stage least squares based iterative identification algorithm for controlled autoregressive moving average (CARMA) systems. *Computers and Mathematics with Applications*.

[78] Wang SJ, Ding R (2013). Three-stage recursive least squares parameter estimation for controlled autoregressive autoregressive systems. *Applied Mathematical Modelling*.

[79] Goodwin GC, Sin KS (1984). *Adaptive Filtering, Prediction and Control*.

[80] Ding F, Chen T (2007). Performance analysis of multi-innovation gradient type identification methods. *Automatica*.

[81] Ding F, Liu PX, Liu G (2010). Multiinnovation least-squares identification for system modeling. *IEEE Transactions on Systems, Man, and Cybernetics B: Cybernetics*.

[82] Ding F, Liu PX, Liu G (2009). Auxiliary model based multi-innovation extended stochastic gradient parameter estimation with colored measurement noises. *Signal Processing*.

[83] Ding F (2010). Several multi-innovation identification methods. *Digital Signal Processing*.

[84] Ding F (2013). Hierarchical multi-innovation stochastic gradient algorithm for Hammerstein nonlinear system modeling. *Applied Mathematical Modelling*.

[85] Ding F, Chen HB, Li M (2007). Multi-innovation least squares identification methods based on the auxiliary model for MISO systems. *Applied Mathematics and Computation*.

[86] Han LL, Ding F (2009). Multi-innovation stochastic gradient algorithms for multi-input multi-output systems. *Digital Signal Processing*.

[87] Wang DQ, Ding F (2010). Performance analysis of the auxiliary models based multi-innovation stochastic gradient estimation algorithm for output error systems. *Digital Signal Processing*.

[88] Xie L, Liu YJ, Yang HZ, Ding F (2010). Modelling and identification for non-uniformly periodically sampled-data systems. *IET Control Theory and Applications*.

[89] Liu YJ, Yu L, Ding F (2010). Multi-innovation extended stochastic gradient algorithm and its performance analysis. *Circuits, Systems, and Signal Processing*.

[90] Liu YJ, Xiao Y, Zhao X (2009). Multi-innovation stochastic gradient algorithm for multiple-input single-output systems using the auxiliary model. *Applied Mathematics and Computation*.

[91] Han LL, Ding F (2010). Parameter estimation for multirate multi-input systems using auxiliary model and multi-innovation. *Journal of Systems Engineering and Electronics*.

[92] Han LL, Ding F (2009). Identification for multirate multi-input systems using the multi-innovation identification theory. *Computers and Mathematics with Applications*.

[93] Ding F, Liu XG, Chu J (2013). Gradient-based and least-squares-based iterative algorithms for Hammerstein systems using the hierarchical identification principle. *IET Control Theory and Applications*.

[94] Ding F, Liu YJ, Bao B (2012). Gradient-based and least-squares-based iterative estimation algorithms for multi-input multi-output systems. *Proceedings of the Institution of Mechanical Engineers, Part I: Journal of Systems and Control Engineering*.

[95] Ding F, Liu PX, Liu G (2010). Gradient based and least-squares based iterative identification methods for OE and OEMA systems. *Digital Signal Processing*.

[96] Ding F (2013). Decomposition based fast least squares algorithm for output error systems. *Signal Processing*.

[97] Liu YJ, Wang DQ, Ding F (2010). Least squares based iterative algorithms for identifying Box-Jenkins models with finite measurement data. *Digital Signal Processing*.

[98] Hu HY, Ding F (2012). An iterative least squares estimation algorithm for controlled moving average systems based on matrix decomposition. *Applied Mathematics Letters*.

[99] Wang DQ, Yang GW, Ding RF (2010). Gradient-based iterative parameter estimation for Box-Jenkins systems. *Computers and Mathematics with Applications*.

[100] Wang DQ (2011). Least squares-based recursive and iterative estimation for output error moving average systems using data filtering. *IET Control Theory and Applications*.

[101] Ding F, Liu G, Liu XP (2010). Partially coupled stochastic gradient identification methods for non-uniformly sampled systems. *IEEE Transactions on Automatic Control*.

[102] Ding F (2013). Coupled-least-squares identification for multivariable systems. *IET Control Theory and Applications*.

[103] Ding F, Chen T (2005). Performance bounds of forgetting factor least-squares algorithms for time-varying systems with finite measurement data. *IEEE Transactions on Circuits and Systems I: Regular Papers*.

[104] Liu YJ, Sheng J, Ding RF (2010). Convergence of stochastic gradient estimation algorithm for multivariable ARX-like systems. *Computers and Mathematics with Applications*.

[105] Ding F, Liu G, Liu XP (2011). Parameter estimation with scarce measurements. *Automatica*.

[106] Liu YJ, Xie L, Ding F (2009). An auxiliary model based on a recursive least-squares parameter estimation algorithm for non-uniformly sampled multirate systems. *Proceedings of the Institution of Mechanical Engineers, Part I: Journal of Systems and Control Engineering*.

[107] Ding J, Han LL, Chen X (2010). Time series AR modeling with missing observations based on the polynomial transformation. *Mathematical and Computer Modelling*.

[108] Ding F, Chen T, Qiu L (2006). Bias compensation based recursive least-squares identification algorithm for MISO systems. *IEEE Transactions on Circuits and Systems II: Express Briefs*.

[109] Ding F, Liu PX, Yang H (2008). Parameter identification and intersample output estimation for dual-rate systems. *IEEE Transactions on Systems, Man, and Cybernetics A: Systems and Humans*.

[110] Ding J, Ding F (2011). Bias compensation-based parameter estimation for output error moving average systems. *International Journal of Adaptive Control and Signal Processing*.

[111] Ding J, Shi Y, Wang H, Ding F (2010). A modified stochastic gradient based parameter estimation algorithm for dual-rate sampled-data systems. *Digital Signal Processing*.

[112] Ding F, Chen T (2004). Least squares based self-tuning control of dual-rate systems. *International Journal of Adaptive Control and Signal Processing*.

[113] Ding F, Chen T (2006). A gradient based adaptive control algorithm for dual-rate systems. *Asian Journal of Control*.

[114] Ding F, Chen T, Iwai Z (2007). Adaptive digital control of Hammerstein nonlinear systems with limited output sampling. *SIAM Journal on Control and Optimization*.

[115] Zhang J, Ding F, Shi Y (2009). Self-tuning control based on multi-innovation stochastic gradient parameter estimation. *Systems and Control Letters*.

[116] Liu YJ, Ding F, Shi Y (2014). An efficient hierarchical identification method for general dual-rate sampled-data systems. *Automatica*.

[117] Ding F (2014). Hierarchical parameter estimation algorithms for multivariable systems using measurement information. *Information Sciences*.

